# The National Institute for the Blind

**Published:** 1922-11

**Authors:** 


### THE NATIONAL INSTITUTE FOR THE BLIND.

The National Institute for the Blind, 224 Great Portland Street, London, W.1, which has just issued its report for 1921-2, is now the largest institution of its kind, not only in the United Kingdom, but throughout the world. Its direct work includes a home for blind babies, a juvenile education department, higher education (at Worcester College for Blind Boys and the Chorley Wood College for Blind Girls), the publication of embossed books, periodicals and music in Braille and Moon type, a School of Massage, the visitation and instruction of the adult blind in their own homes, the maintenance of homes and hostels for the aged blind, and the relief of the blind poor. Unless additional funds are immediately forthcoming, very serious retrenchment of activities will be necessary.

It has always been the policy of the Institute to work with other institutions. At the beginning of last year, in pursuance of this policy, the eight leading workshops and institutions in London were invited to co-operate in raising money for the many urgent requirements of the 7,000 blind in the metropolitan area, and the National Institute is now conducting the Greater London Fund for the Blind, with the assistance of an Advisory Committee. The Fund has been in existence for 15 months, and up to March 31 last has distributed the sum of £20,800. During the year grants amounting to £40,740 were made to local institutions for the blind in all parts of the country.

It is important that the specialised training of blind babies should be taken in hand from the first year of their existence. "Sunshine House" was established by the Institute at Chorley Wood, Herts, in 1918, as a home for blind babies under five years of age. The home has always been full, with a long waiting list. If another "Sunshine House" can be opened in the north of England, the Institute believes that the problem of the blind child will be on the way to solution.

The Institute has been described as the biggest publishing house for the blind in the world. It makes ample allowance for different tastes and requirements. Poetry, essays, textbooks and works on the arts and sciences, history and biography are to be found side by side with the latest novels, short stories and current literature. Fourteen Braille magazines and newspapers, one magazine in Moon type, and a letterpress magazine, the "Beacon," are also published. The "Braille Mail" is a weekly newspaper, giving a brief but full day-by-day summary of the world's news. All embossed books are sold at a 75 per cent. discount on cost price to the blind in this country, at a 50 per cent. discount to the blind in the British colonies, and at cost price to the blind in foreign countries. The Manuscript Department supplies books of an educational or special character to blind students training for the professions. The Music Department provides music in Braille. The catalogue includes not only the best classics, but the latest piano and organ pieces, songs and dance music. The music for standard examinations has been much in demand, and the organ in the Armitage Hall of the National Institute, a model of the organ at the Royal College of Organists, is always available to students who wish to practise for the College examinations.

One hundred and fifty-two students have been trained for the recognised massage examinations, and all have qualified; the majority have also passed the examination in remedial gymnastics and the medical electricity examination. Substantial financial help is rendered by the Institute in respect of students' maintenance, fees, textbooks and services of a guide. A special library of massage literature in embossed type is at the free disposal of students and graduates. Through the After-Care Section posts are secured for students after qualifying, and assistance has been rendered to enable them to build up private practices; in a number of instances apparatus has been supplied. The Institute has trained three hospital nurses whose sight became so affected that it was impossible for them to continue their occupation. Two have been settled in hospital posts; the third has started in private practice.

The Home-Teaching Branch visits the blind in their own homes, teaching them to read Braille and Moon, giving instruction in various useful handicrafts, and advising and assisting them in every way, especially those who have recently lost their sight. Thirty-six teachers, all, with one exception, blind, have visited 5,542 people, making a total of 71,062 visits during the year. A feature of this branch's work is the regular visitation of the blind in workhouses and infirmaries.

During the year the number of new cases dealt with was 963, making a total now on the Institute's register of 7,039 cases. Though confronted with abnormal periods of distress, a sum of £11,371 17s. 4d. has been distributed in relief. Gifts to the value of £989 5s. 9d. have been provided, and dental and surgical requisites supplied to 35 people. The After-Care Department is responsible for 140 persons who are being educated or who are undergoing training in industrial and professional occupations. Educational fees paid during the period under review amounted to £1,250 10s., and training ees to £3,492 7s. 11d.

---

The annual report of the Association of Certificated Blind Masseurs shows that its efforts to secure recognition and status for its members have met with considerable success. Under a new arrangement, the Association receives a definite grant from St. Dunstan's and the National Institute for the Blind. The total membership to date is 168, 95 of these being blinded soldiers, 34 civilian masseurs and 39 masseuses All members are fully qualified and hold the recognised certificates of the profession. The official organ, the "Massage Journal," is published monthly in embossed type, and a special feature is made of lectures and articles, not only on massage, but on medical and surgical matters in general. The Council hopes that the services of its members will be utilised in future by medical men to a greater extent even than heretofore. The late Sir Arthur Pearson was succeeded as President in January, 1922, by Sir Robert Jones, K.B.E., C.B.E., F.R.C.S.

### ARMISTICE MONUMENT.

The King has notified the Ligue des Chefs de Section et des Soldats Combattants (the French ex-Service men's union) that he gives with pleasure his patronage to the ceremony of the unveiling of the Armistice Monument in the Forest of Compiègne on November 11. The monument is being erected on the spot where the Germans signed Marshal Foch's armistice terms, and will be inscribed: "Here, on November 11, 1918, succumbed the criminal pride of the German Empire." A list of the names of those who have subscribed to the monument fund will be sealed under the stone by Marshal Foch in the course of the ceremony, and in order that this list may be ready early, intending subscribers are requested to send their donations as soon as possible to M. le Capitaine Ternisien, Secrétaire Général de la Ligue des Chefs de Section et des Soldats Combattants, 248, Rue du Faubourg Saint-Honoré, Paris (8e).

### RUBBER GLOVES.

Apart from surgery, rubber gloves are now widely used in manufactures and trades, such as tanning and dyeing, in the handling and packing of chemical substances injurious to the skin, and in the rougher kinds of domestic work. It is claimed for the "Eclipse" household gloves, which are of seamless red rubber, that they will outlast three or four pairs of ordinary quality. Strong, supple and well finished, they can be sterilised without injury by boiling or with antiseptics. They are sold by all stores and chemists in standard sizes from 6 to 10.

### THE "RELAY" AUTOMATIC TELEPHONE SYSTEM.

Hospitals and other large institutions which are not satisfied with their existing inter-departmental telephone arrangements would do well to investigate the system of the Relay Automatic Telephone Co., Ltd., by which numbers are called and disconnected without the help of a human operator. Switchboards from ten lines upwards can be supplied, and the General Post Office will now instal the system on rental for private branch exchanges, combining automatic communication between departments and connection to the public exchange service. An admirably produced catalogue can be obtained on application from the company at Marconi House, Strand, London, W.C. 2.

